# Multicellular tumor spheroid model to evaluate spatio-temporal dynamics effect of chemotherapeutics: application to the gemcitabine/CHK1 inhibitor combination in pancreatic cancer

**DOI:** 10.1186/1471-2407-12-15

**Published:** 2012-01-13

**Authors:** Isabelle Dufau, Céline Frongia, Flavie Sicard, Laure Dedieu, Pierre Cordelier, Frédéric Ausseil, Bernard Ducommun, Annie Valette

**Affiliations:** 1 USR 3388 CNRS/Pierre Fabre, Centre de Recherche et Développement Pierre Fabre, 3 avenue Hubert Curien BP 13562, 31035 Toulouse Cedex1, France; 2 Université de Toulouse and CNRS, ITAV-UMS3039, Toulouse, France; 3 CHU de Toulouse, Toulouse, France; 4 INSERM and Université de Toulouse, CRCT-UMR1037, Toulouse, France

**Keywords:** Tumor spheroid, Combination, Gemcitabine, CHK1 inhibitor, Pancreatic cancer

## Abstract

**Background:**

The multicellular tumor spheroid (MCTS) is an in vitro model associating malignant-cell microenvironment and 3D organization as currently observed in avascular tumors.

**Methods:**

In order to evaluate the relevance of this model for pre-clinical studies of drug combinations, we analyzed the effect of gemcitabine alone and in combination with the CHIR-124 CHK1 inhibitor in a Capan-2 pancreatic cell MCTS model.

**Results:**

Compared to monolayer cultures, Capan-2 MCTS exhibited resistance to gemcitabine cytotoxic effect. This resistance was amplified in EGF-deprived quiescent spheroid suggesting that quiescent cells are playing a role in gemcitabine multicellular resistance. After a prolonged incubation with gemcitabine, DNA damages and massive apoptosis were observed throughout the spheroid while cell cycle arrest was restricted to the outer cell layer, indicating that gemcitabine-induced apoptosis is directly correlated to DNA damages. The combination of gemcitabine and CHIR-124 in this MCTS model, enhanced the sensitivity to the gemcitabine antiproliferative effect in correlation with an increase in DNA damage and apoptosis.

**Conclusions:**

These results demonstrate that our pancreatic MCTS model, suitable for both screening and imaging analysis, is a valuable advanced tool for evaluating the spatio-temporal effect of drugs and drug combinations in a chemoresistant and microenvironment-depending tumor model.

## Background

Pancreatic ductal adenocarcinoma (PDAC) is one of the most lethal cancers with less than 5% of overall patient survival after 5 years. Local and distant invasion, resistance to chemotherapy and radiotherapy and lack of early detection are responsible for this poor prognosis. Gemcitabine (2',2'-difluorodeoxycytidine, a pyrimidine nucleoside analogue) chemotherapy, is the standard treatment of the patients. The combination of gemcitabine with other chemo- or biotherapies has resulted in a very limited prognostic improvement. Recently, a high throughput RNAi screen identified the checkpoint kinase 1 (CHK1) as a gene conferring resistance to gemcitabine in pancreatic cancer cells [1]. CHK1 is a key component of the cell cycle checkpoints that are activated by genomic and replicative stress (review in [2]). This checkpoint activation is known to facilitate DNA repair. Consequently, CHK1 may play an important role in the resistance of tumor cells to genotoxic therapy, raising the possibility that inhibitors of checkpoint kinases may be useful adjuvant agents in chemotherapy of cancer. In the case of pancreatic cancer, in vitro and in vivo studies have shown that CHK inhibitors enhance the antitumor activity of gemcitabine [3-5].

The MultiCellular Tumor Spheroid (MCTS) model is generally considered as a better model than two dimensional culture to predict the in vivo response to drug treatments [6-8] and it is now widely accepted that MCTS reproduce more accurately the tumor microenvironment than monolayer cell cultures. While growing, spheroids display a gradient of proliferating cells from the outer cell layers with quiescent cells located more centrally. When deprived of oxygen and glucose, central cells die and a necrotic zone is formed. This cell heterogeneity is similar to that found in avascular micro-regions of tumors [9]. It is well established that solid tumor environment induces the level of drug resistance to many chemotherapeutic agents. This phenomenon, called multicellular resistance [10], emerges as soon as cancer cells have established contacts with surrounding cells or extracellular matrix, i.e. its microenvironment. In MCTS, cancer cells can acquire this multicellular resistance by interacting efficiently in 3 dimensions with their environment [10-12].

In order to contribute to the discovery of new anti pancreatic cancer agents or new potent combinations with gemcitabine, we describe here the development and the validation of a new spheroid model mimicking the structure and chemo resistance of pancreatic solid tumors compared to conventional 2D cell culture models. We also present the spatio-temporal parameters of the biological response of gemcitabine alone or combined with a CHK1 inhibitor, CHIR-124.

## Materials and methods

### Reagents

Gemcitabine was purchased from Sigma. CHIR-124 was a generous gift of Dr Alain Pierré (Institute de Recherche Servier).

### Cell culture

Capan-2 pancreatic cancer cells were cultured in DMEM/F12 (Invitrogen, France) containing 10% FCS with 2 mmol/l glutamine and penicillin/streptomycin in a humidified atmosphere of 5% CO2 at 37°C. Capan-2 cells were transduced with a lentiviral vectors coding for fused green -emitting fluorescent proteins to Geminin [13].

### Spheroid generation

Spheroids were prepared according to [14]. A Capan-2 cell suspension containing 10^4 ^cells/ml of DMEM/F12 supplemented with EGF (20 ng/ml) (Invitrogen) and B27 (Invitrogen) was prepared. 100 μl of this cell suspension were plated on each well of poly-HEMA-coated 96-well plates. The plates were centrifugated at 200 g during 6 min and then incubated in a humidified atmosphere of 5% CO2 at 37°C. By using this technique we obtained single spheroids in each well, the variation of size between spheroids is less than 10%. In order to generate quiescent spheroids, after a first 4 days growth phase in defined medium (DMEM/F12 supplemented with EGF and B27), spheroids were washed twice with media containing 10% FCS, and then incubated with this media during 1-6 days.

### Spheroid viability quantification

Spheroid viability was quantified by ATP monitoring with the Perkin Elmer ATPlite™ assay system. This system is based on the production of light caused by the reaction of ATP, a cell viability marker present in cell lysate, with added luciferase and D-luciferin. We adapted ATPlite assay procedure for spheroid application, especially concerning spheroid dissociation and cell lysis. Then 100 μl of mammalian cell lysis solution (ATPlite kit) were added to each well containing one spheroid in 100 μl of culture medium. The plate was shaken for 20 min. In order to read luminescent signal, 75 μl of the cell lysate was transferred to a black 96-well plate. Then 37 μl of DMEM/F12 medium containing 10% FCS and 37 μl of ATPlite kit substrate solution were added. After 15 min of shaking, the luminescence signal was read on an Envision^® ^plate reader (Perkin Elmer).

### Immunofluorescence on frozen sections

Capan-2 spheroids were rinsed with PBS and fixed in 4% neutral-buffered formalin (Sigma) for 2 h. After fixation, spheroids were processed for 5 μm frozen sections. Sections were incubated overnight at 4°C with antibodies directed against cleaved form of PARP (rabbit monoclonal, Epitomics, 1/1000), or γH2AX phosphorylated (mouse monoclonal, Upstate, 1/200) and Ki67 (rabbit polyclonal, Santa Cruz, 1/200). After washing in PBS/Triton 0.1% v/v, the secondary antibody was applied (Alexa 488-anti-mouse or Alexa 594-anti-rabbit, Molecular Probes, 1/800, for 1 h at room temperature). To determine cell cycle repartition, sections of Capan-2 spheroids expressing the green FUCCI probe were directly analyzed by fluorescence imaging. The observations were based on the examination of 3 sections from at least 5 spheroids. Each experiment has been repeated a minimum of 3 times.

### Cytotoxicity assays

Spheroids were generated using 1000 cells in 100 μl per well as indicated in spheroid generation section. After 4 days of culture, chemotherapeutic agents or combinations were added (10 μl/well). Spheroid viability was evaluated by ATP quantification after 72 h compound treatment. Tests were performed in triplicate and the data presented are from at least three separate experiments. ATP content percentage was calculated with regard to non-treated spheroid and showed cell growth inhibition and/or toxicity. The 50% effective concentration (EC50) of a compound is the concentration which provokes 50% of the maximal effect (ATP production decrease) of this drug. Curve fittings were performed with GraphPad (San Diego, CA) Prism version 4.0 software using the sigmoidal dose-response to determine EC50 values.

## Results

### Generation of Capan-2 spheroids in microplate

**Figure 1:**
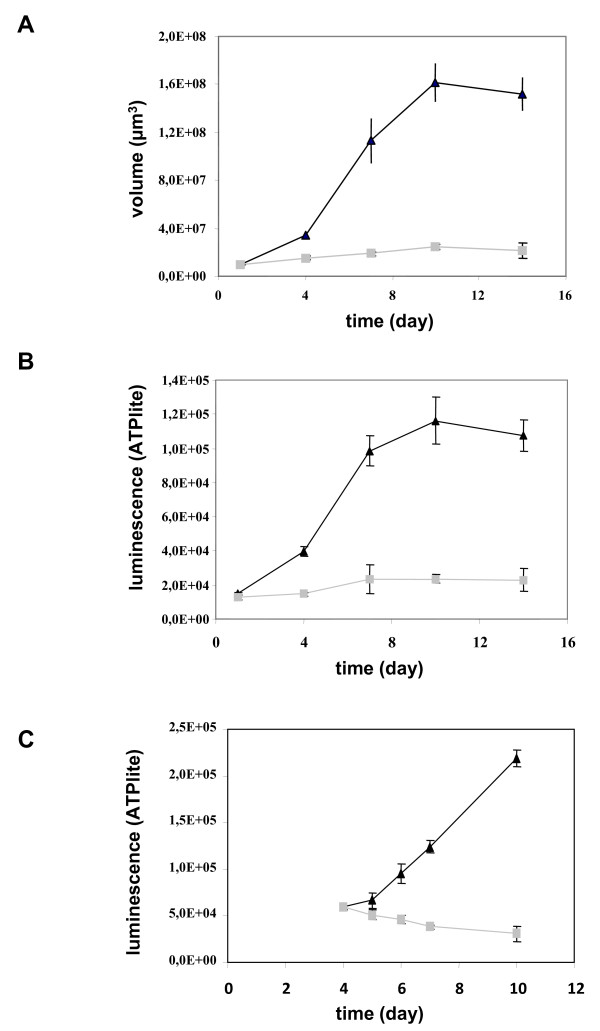
**Growth of Capan-2 spheroids**. The Capan-2 spheroids were grown in the presence of 10% serum (gray squares) or defined medium added with EGF and B27 (black triangles) without any medium change (**a **and **b**). (**a**) Spheroid size. The diameter of each spheroid was measured by microscopic observation with an ocular micrometer and sphere volume was calculated. (**b**) Cell viability. Spheroid viability was quantified by ATP monitoring with the luminescent ATPlite assay. (**c**) Induction of a quiescent state on Capan-2 spheroid. Capan-2 spheroid culture was initiated in presence of 10% serum and EGF and four days after culture initiation, EGF was (gray squares) or not (black triangles) removed from the culture medium. Capan-2 spheroid viability was followed during a 6 days additional period with the luminescent ATPlite assay. Each point is the mean ± SD of 6 spheroids.

In order to obtain a model of pancreatic multicellular spheroid, we tested several pancreatic cancer cell lines including BxPC3, MiaPaCa, Panc-1, AsPC-1, Capan-2. A pancreatic cancer spheroid model was obtained only with Capan-2 cell line. Seeding of 10^3 ^Capan-2 pancreatic cancer cells in DMEM/F2 medium supplemented with 10% serum allowed cell association and stabilization in spherical structure after centrifugation. However, whereas this medium allowed Capan-2 cell proliferation in monolayer culture, it was not able to sustain Capan-2 cell growth in spheroid in 96-well plates (Figure 1a). Consequently, different growth media composition were evaluated and we found that defined DMEM/F12 medium supplemented with EGF and B27 induced Capan-2 spheroid growth up to 16-fold between day 1 and day 10. Determination of cell viability by measurement of cell ATP content (Figure 1b) confirmed that Capan-2 spheroids grown faster in the defined medium. Intra- and inter-assay precision of spheroid volume and ATP measurement was found to be suitable to ensure robust pharmacological studies (variation of size and ATP were less than 10%) (data not shown). To confirm the dependence on EGF, Capan-2 spheroids were cultured in defined medium supplemented with EGF. Four days later, EGF was washed out and Capan-2 spheroids were maintained in 10% serum. In this condition, we observed that Capan-2 spheroid growth was inhibited (Figure 1c).

**Figure 2:**
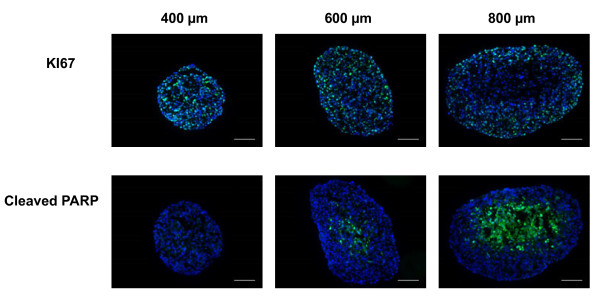
**Distribution of proliferative and apoptotic cells in Capan-2 spheroids of different sizes**. Ki-67 and cleaved form of PARP were analyzed by immunodetection on 5 μm frozen sections in Capan-2 spheroids of different size. 400, 600 and 800 μm correspond respectively to spheroid at days 4, 7, and 12 after culture initiation in defined medium supplemented with EGF and B27. The scale bar corresponds to 100 μm. Results shown are representative of the examination of 3 sections from 5 spheroids. Each experiment has been repeated 3 times.

The spheroid internal structure depends on a nutrient and oxygen gradient which controls a decreasing gradient of cell proliferation from the periphery to the center of spheroid. A central necrotic area is generally observed in spheroids larger than 500 μm due to critical O_2 _concentration in the central zone [9]. We determined the repartition of proliferative and apoptotic cells in Capan-2 spheroids of various sizes cultured in defined medium supplemented with EGF and B27 (Figure 2). Formalin-fixed tissue-teck-embedded Capan-2 spheroid sections were immuno-stained for the proliferation and apoptotic markers Ki-67 and cleaved PARP respectively. We found that proliferative and non proliferative cells were distributed throughout the 400 μm size Capan-2 spheroid and a gradient of proliferation appears on spheroid measuring 600 μm and more in diameter. While apoptosis was not detected in 400 μm spheroids, apoptotic cells were observed in the center of the spheroid of larger diameters (Figure 2). Consequently, this model allows the investigation of drug response taking into account cell heterogeneity.

### Resistance to gemcitabine treatment of the Capan-2 spheroid model

**Figure 3:**
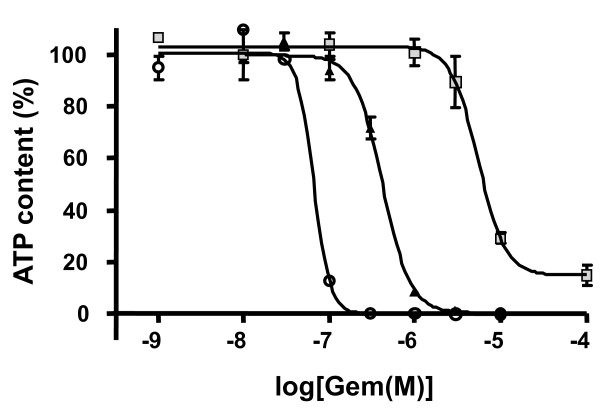
**Gemcitabine effect on cell viability after 72 h treatment on Capan-2 cells cultured as monolayer (open circle), spheroid in defined medium supplemented with EGF and B27 (black triangles), or as quiescent Capan-2 spheroid (gray squares)**. Each point is the mean ± SD of 3 replicates. Dose-response experiments were fitted with GraphPad Prism software.

Considering increase in spheroid size, change in proliferation gradient and the occurrence of a necrotic core, we applied cytotoxic treatment (3 days) between days 4 and 7, thus avoiding overlapping effects. Indeed, we did not observe significant difference in gemcitabine EC50 between 4, 5, 6 and 7 days spheroids (diameter ranging from 400 to 600 μm) (data not shown). As a consequence we cultured spheroids for four days before treatment as this protocol is compatible with automated HTS application. We first compared the effect of gemcitabine on Capan-2 cells growing as monolayer and as spheroid. Figure 3 shows the effect of different gemcitabine concentrations on spheroid culture compared to the monolayer culture. We observed that a 3 day treatment with gemcitabine exerted a similar efficiency (100% ATP decrease) but gemcitabine potency was found to be much higher in monolayer culture (EC50 = 0.66 10^-7 ^M) compared to spheroids (EC50 = 4.2 10^-7 ^M) indicating that gemcitabine effect could be correlated to multicellular growth condition.

To evaluate if this resistance is linked to the presence of quiescent cells in the Capan-2 spheroid, we tested the response to gemcitabine treatment of quiescent spheroids. Capan-2 spheroid need for EGF was used to induce a quiescent state. As already shown in Figure 1c, when Capan-2 spheroids were cultured in absence of EGF in 10% serum, an inhibition of growth was observed. In this condition the potency of gemcitabine was 13-fold lower in quiescent Capan-2 spheroid than in proliferative Capan-2 spheroid (Figure 3). Thus this Capan-2 spheroid model mimics multicellular resistance to gemcitabine.

### Gemcitabine cytotoxicity in capan-2 spheroid is associated with induction of DNA damage, cell cycle perturbation and apoptosis

**Figure 4:**
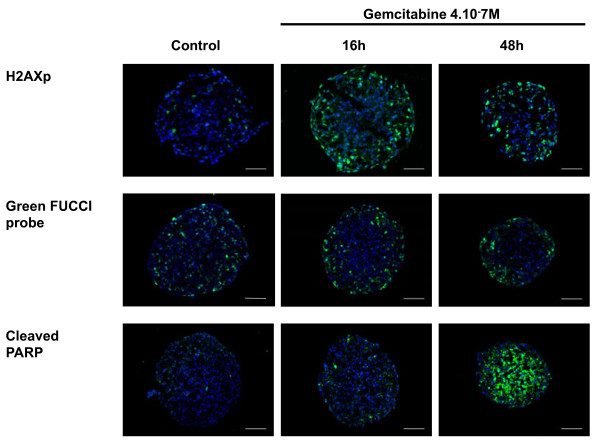
**Spatio-temporal response of Capan-2 spheroid to gemcitabine**. Analysis of gemcitabine response was done on 5 μm frozen sections of Capan-2 spheroids treated 16 h and 48 h with 4.10^-7 ^M gemcitabine. DNA damage was revealed by immunodetection of phosphorylated of γH2AX, S phase checkpoint was monitored on Capan-2 spheroid expressing the geminin-mAG FUCCi green probe and apoptosis was analyzed by immunodetection of cleaved form of PARP. Images were collected using a X10 objective. The scale bar corresponds to 100 μm. Results shown are representative of the examination of 3 sections from 5 spheroids. Each experiment has been repeated 3 times.

The gemcitabine cytotoxic effect is mediated by induction of DNA damage [15]. We used the spheroid model to determine how gemcitabine-induced DNA damage occurs in function of cell position within the spheroid. The Histone H2AX phosphorylation at Ser139 (γ-H2AX) was used as a marker of DNA damage. Immunodetection of this phosphorylated form γ-H2AX on frozen sections of gemcitabine-treated Capan-2 spheroids (used at their EC50 value, 4.10^-7 ^M) showed that DNA damage was restricted to the outer cell layer until 48 h after gemcitabine addition (Figure 4).

In order to monitor gemcitabine-induced cell cycle intra-S and G2/M checkpoints response in a 3-D context we used Capan-2 cells expressing FUCCI reporter corresponding to the fluorescent protein geminin-mAG (green) which accumulates in cell nuclei in S-, G2- and M-phase [13]. In control spheroids the FUCCI-green reporter was expressed in cells located throughout the spheroid however the proportion of FUCCI-green cells was higher in cells located in the outer cell layer (Figure 4). In agreement with the fact that a S-phase checkpoint is activated in response to gemcitabine [16], a 16 h treatment of Capan-2 spheroid with gemcitabine resulted in a regionalization of the FUCCI green expressing cells that located only in the outer cell layers. This accumulation of cells in the S/G2/M phases of the cell cycle was maintained 48 h after gemcitabine addition.

The therapeutic potential of gemcitabine results from its ability to induce apoptosis in tumor cells. Gemcitabine-induced apoptosis was examined using immunodetection of cleaved form of PARP on frozen sections. We found that, whereas apoptotic cells were not detected 16 h after addition of gemcitabine, a massive apoptosis occurred throughout the spheroid after 48 h of treatment (Figure 4).

### Gemcitabine and CHIR-124 induced a synergistic cytotoxic effect on capan-2 spheroid

**Figure 5:**
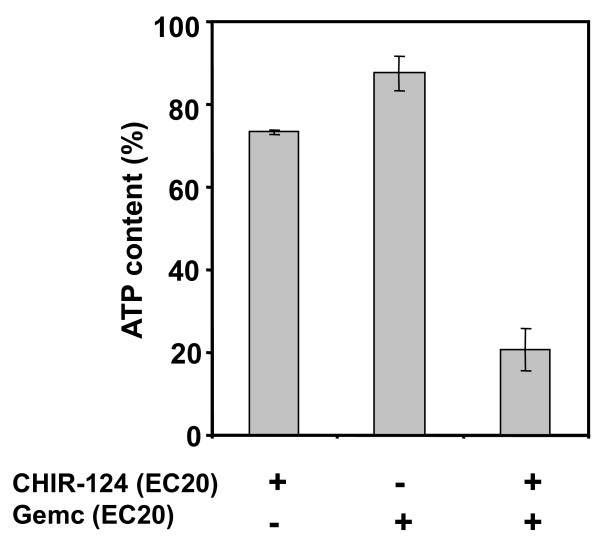
**Gemcitabine and CHIR-124 produce a synergistic cytotoxic effect on Capan-2 spheroid**. Spheroids were treated for 72 h after 4 day initial culture with gemcitabine, CHIR-124 or a combination of the 2 compounds at concentration corresponding to their respective EC20 values. Spheroid viability was quantified at day 7 by ATP monitoring with the luminescent ATPlite assay. ATP content percentage was calculated with regard to non-treated spheroid and showed cell growth inhibition and/or toxicity.

Inhibitors of CHK1 have previously been shown to enhance gemcitabine cytotoxic effect against pancreatic cancer cells [3-5]. CHIR-124 is a potent inhibitor of CHK1 activity [17]. CHIR-124 induced a decrease in Capan-2 spheroid viability (EC50 = 6.7 10^-8 ^M; 100% efficiency) (data not shown). We then determined the impact of CHIR-124 on the sensibility of Capan-2 spheroid to gemcitabine. For combination experiments we selected doses of CHIR-124 and gemcitabine below their respective EC50. For several CHIR-124/Gemcitabine combinations, we observed a synergistic effect of the two compounds corresponding to higher inhibition potency than the addition of the two compounds tested separately. For example, a co-treatment of Gemcitabine and CHIR-124 at their EC20 resulted in a 79% ATP decrease. Thus, at a sub-toxic concentration, CHIR-124 potentiated the cytotoxic effect of a low dose of gemcitabine (Figure 5).

### CHIR-124 modulates the spatial response of capan-2 spheroid to gemcitabine

**Figure 6:**
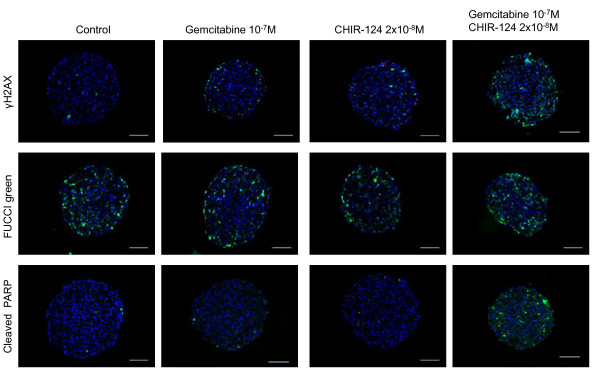
**Spatio-temporal response of Capan-2 spheroid to gemcitabine and Chk1 inhibitor**. Analysis of gemcitabine response in presence and absence of CHK1 inhibitor was done on 5 μm frozen sections of Capan-2 spheroids treated 16 h with 10^-7 ^M gemcitabine alone, 2 10^-8 ^M CHIR-124 alone or the combination of gemcitabine and CHIR-124 at these same doses. DNA damage was revealed by immunodetection of phosphorylated of γH2AX, S phase checkpoint was monitored on Capan-2 spheroid expressing the geminin-mAG Fucci green probe and apoptosis was analyzed by immunodetection of cleaved form of PARP. Images were collected using a X10 objective. The scale bar corresponds to 100 μm. Results shown are representative of the examination of 3 sections from 5 spheroids. Each experiment has been repeated 3 times.

We tested whether the CHIR-124 potentiation of gemcitabine cytotoxic effect on Capan-2 spheroid correlates with an increase in gemcitabine-induced DNA damage. As shown in Figure 6 (after a 16 h treatment), a low gemcitabine concentration had minimal effect on the induction of DNA damage (γH2AX staining), apoptosis (cleaved PARP) and accumulation of cells in S/G2/M phases (FUCCI reporter) in the outer cell layer. CHIR-124 at a low dose showed a very weak effect on cell accumulation in the S/G2/M phase, apoptosis and DNA damage. When CHIR-124 was combined with gemcitabine at these same doses, the effect on DNA damage and apoptosis was significantly improved mainly in the outer layer of the MCTS. All these measurements were performed in several independent experiments as shown for apoptosis image analysis (Additional file 1: Figure S1). From these images, induced apoptosis was quantified by counting PARP-C positive cells per spheroid section and showed a clear synergic effect (Additional file 2: Figure S2).

It has been reported that CHK inhibitors abrogate the gemcitabine induced S phase checkpoint [3-5]. However, in our combination experiments, the gemcitabine-induced accumulation of cells in S/G2/M phases is too low to observe a clear reversion of this effect in presence of CHIR-124 (Figure 6). These results indicated that the potentiation of spheroid proliferation inhibition of gemcitabine by CHIR-124 was associated with a cell cycle checkpoint abrogation leading to the induction of DNA damage and apoptosis.

## Discussion

Standard chemotherapeutic drugs have limited effect in large-scale clinical trials for pancreatic cancer. Because of the very poor prognosis of this type of cancer, novel approaches are therefore urgently needed. Most in vitro screening approaches are based on monolayer culture of pancreatic cancer cells but it is well established that tumor microenvironment plays an important role in response to chemotherapy. It is therefore of major importance that more predictive pharmacological models be developed for the assessment of new therapeutic strategies.

Multicellular Tumor Spheroids are of particular interest as they offer a level of intermediate complexity that recapitulate the three-dimensional organization of a tumor and integrate the notion of microenvironment. The production of 500-600 μm large spheroids from various epithelial cancer cell lines has already been shown for colon, breast, prostate and kidney but not pancreas with the liquid overlay technology [18]. Spheroids from several pancreatic ductal adenocarcinoma (PDAC) cell lines were obtained on micro-patterned culture plates but no pharmacological analysis were presented with these models [19]. Recently, PDAC cell lines grown in 3D collagen microenvironment were shown to proliferate in the presence of gemcitabine whereas they stopped growing when cultivated on tissue culture plastic indicating that 3D cell organisations could have an impact on pancreatic cancer cell drug sensitivity [20]. Then, the development of new MCTS models represents an interesting way to improve the discovery of new treatment. By using the in vivo validated gemcitabine and CHIR124 molecules [3], we show here that our Capan-2 MCTS model for pancreatic cancer could detect effective drug combinations.

In this study we developed an "automation friendly" spheroid model of Capan-2 pancreatic cancer cell spheroids in 96 well-plates. We chose ATP quantification to measure the effect of chemical compounds on cell viability and proliferation. We showed that epidermal growth factor (EGF) was necessary to maintain Capan-2 cell proliferation in a 3-D context, whereas it was not the case in monolayer. It is well known that EGF plays an important role in pancreatic cancer progression and EGF and its ligand over-expression have been frequently observed in pancreatic cancer [21,22]. A recent study reporting the effects of EGF ligands in different culture conditions of ovarian cancer cells clearly showed that in contrast to monolayer culture, spheroids facilitated growth stimulatory activity of EGF ligands [23]. This EGF dependent-proliferation of spheroids emphasized the relevance of this model by comparison with cell monolayer and with tumor context. Moreover, the EGFR systems and associated signaling pathway could be promising targets for pancreatic cancer treatment [24]. Consequently Capan-2 cell spheroid appears to be a relevant model to screen for EGF signaling targeting compounds.

A proliferation gradient was observed for spheroids around 600 μm diameter: proliferative cells were located in the outer layer whereas quiescent cells were located more centrally. It has been previously shown that when the central cells become deprived of oxygen and glucose, cell death and necrosis occur [9]. According to this, we found that apoptotic cells were detected in the spheroid center after 7 days when the spheroid size reached 600 μm. This proportion greatly increased until day 12. The characterization of the proliferation gradient in the spheroid of different sizes clearly showed that there was a window to test antitumoral compounds. This window started when proliferation gradient was established (after 4 days) but before central necrosis appeared at onset of treatment (before 7 days).

Most in vitro studies on the response of pancreatic cancer cell to gemcitabine were based on monolayer cell culture. A study reports that gemcitabine was less potent when cancer cells were grown as multilayer compared to monolayer cultures [25]. It is well established that for many chemotherapeutic drugs a solid tumor environment results in an increased level of drug resistance, a phenomenon called the multicellular resistance. Multicellular resistance emerges as soon as cancer cells have established contacts with their microenvironment, homologous cells, heterologous cells or extracellular matrix [10,26]. This contact dependent resistance can be observed when cell are cultured as spheroid. Spheroid culture of glioblastoma cells are less sensitive to gemcitabine than monolayer cells [27]. Our results show that pancreatic Capan-2 cells cultured as spheroids are also less sensitive to gemcitabine than Capan-2 monolayer. This result agrees with a recent study showing that a 3-D collagen microenvironment protects pancreatic cancer cells from gemcitabine-induced proliferation arrest [20]. Spheroid permeability, presence of quiescent and hypoxic cells could explain this resistance [10]. Our observation that gemcitabine potency was reduced in quiescent Capan-2 spheroid suggests that pancreatic cancer cell proliferation status plays a role in gemcitabine response.

DNA damage induced by gemcitabine results in activation of S cell cycle checkpoint and apoptosis [15]. In addition to assess the global cytotoxicity of anticancer agents, the spheroid model allows to image cell response in function of their position within the spheroid [12,28]. H2AX phosphorylation, which has been demonstrated as a pharmacodynamic indicator of gemcitabine-induced stalled replication forks [15], was first used to image gemcitabine response in Capan-2 spheroid. The establishment of gemcitabine-induced S phase checkpoint was characterized by using Capan-2 cells expressing the Fucci reporters corresponding to the fluorescent protein geminin-mAG (green) that is expressed in S/G2/M phases of the cell cycle. Our results show that 16 h after gemcitabine addition only the cells located in the outer cell layer are targeted by gemcitabine. Indeed, cells of the outer cell layer are those with damaged DNA and accumulated in the S/G2/M phases of the cell cycle. This spatially confined DNA damage may result from limited drug penetration or a low sensitivity of non-proliferating cells in deeper spheroid layers. Our results do not discriminate between these two hypotheses. One limitation to gemcitabine efficacy is its poor penetration in human tumors [22,29,30]. Using a multicellular layer method to study drug penetration it has been shown that the penetration of gemcitabine in multicellular cell layer is independent of cell concentration but decrease with the thickness of the layer [31]. 48 h after gemcitabine addition, cells arrested in the S phase remained located in the outer cell layer whereas DNA damages and apoptosis were detected throughout the spheroid suggesting that DNA damage rather than cell cycle arrest are correlated with apoptosis. This result agrees with a previous study showing that in spheroids the persistence of DNA damage determined by γH2AX staining predicted clonogenic cell survival [32].

One field of spheroid interest is the study of drug combination. Inhibition of CHK1 represents a targeted approach to selectively enhance the cytotoxicity of DNA-damaging agents in tumor cells. Whereas, p53-deficient cells have been preferentially killed by the combination of a DNA damaging agent, which arrest the cell cycle in G2, followed by CHK1 inhibitor, p53-proficient tumors could potentially be targeted by concurrent administration of an antimetabolite and a CHK1 inhibitor [33]. CHK1 inhibitors are able to potentiate gemcitabine cytotoxicity in vitro and in vivo [3,4,16,34,35]. In agreement with these results our data show that gemcitabine and a CHK1 inhibitor (CHIR-124) exert a synergistic cytotoxic effect on Capan-2 spheroid. This synergic cytotoxic effect was associated with an increase in the ability of gemcitabine to trigger DNA damage and apoptosis. Taken together these data indicate that the spheroid model provide new information concerning the role of cancer cell microenvironment on the gemcitabine and CHK1 inhibitor pancreatic cancer cell response.

## Conclusions

In conclusion we have developed a 3-D model of pancreatic cancer cells growing as single spheroid with homogenous size in 96 -well plates. The rationale for using spheroid model in high throughput anticancer drugs screening has been highlighted [8,35]. The Capan-2 spheroid model may be useful in evaluating compounds targeting the EGF signaling pathway. Furthermore our study underscores the predictive power of three-dimensional culture to assess the pharmacological activity of drug combination. While this model does not really mimic in vivo situation, particularly for compound delivery and stability, this model may partly reduce animal xenograft utilization. Pancreatic cancer spheroid model could therefore serve as an intermediate decision-making step in the pre-clinical development of drug combination for pancreatic cancer.

## Competing interests

The authors declare that they have no competing interests.

## Authors' contributions

ID, CF, FS, LD, PC and AV performed experiments. FA, BD and AV directed the study. ID, FA, BD and AV wrote the paper. All authors read and approved the final manuscript.

## Pre-publication history

The pre-publication history for this paper can be accessed here:

http://www.biomedcentral.com/1471-2407/12/15/prepub

## Supplementary Material

Additional file 1 **Figure S1**. Induction of apoptosis upon exposure of Capan-2 spheroid to Gemcitabine and CHIR-124. Analysis was performed as described in Figure 6. Apoptosis was revealed by immunodetection of cleaved form of PARP. Three sections from different spheroids are shown here to illustrate the reproducibility of the observation. The scale bar corresponds to 100 μm. Click here for file

Additional file 2 **Figure S2**. Quantification of the induction of apoptosis upon exposure of Capan-2 spheroid to Gemcitabine and CHIR-124. Analysis was performed as described in Figure 6. Apoptosis was revealed by immunodetection of cleaved form of PARP. Spheroid sections were analyzed to quantify the number of cells with cleaved PARP using the FIJI software (measure plugin). The data are expressed as the percentage of spheroid sections displaying 0 to 10, 10 to 20, or more than 20 PARP-C positive cells. Click here for file
