# Supplementary figures and images for: Multicellular tumor spheroid model to evaluate spatio-temporal dynamics effect of chemotherapeutics: application to the gemcitabine/CHK1 inhibitor combination in pancreatic cancer
Source: BMC Cancer. 2012 Jan 13;12:15. doi: 10.1186/1471-2407-12-15 (PMC3280152; doi:10.1186/1471-2407-12-15)

## Slide 1
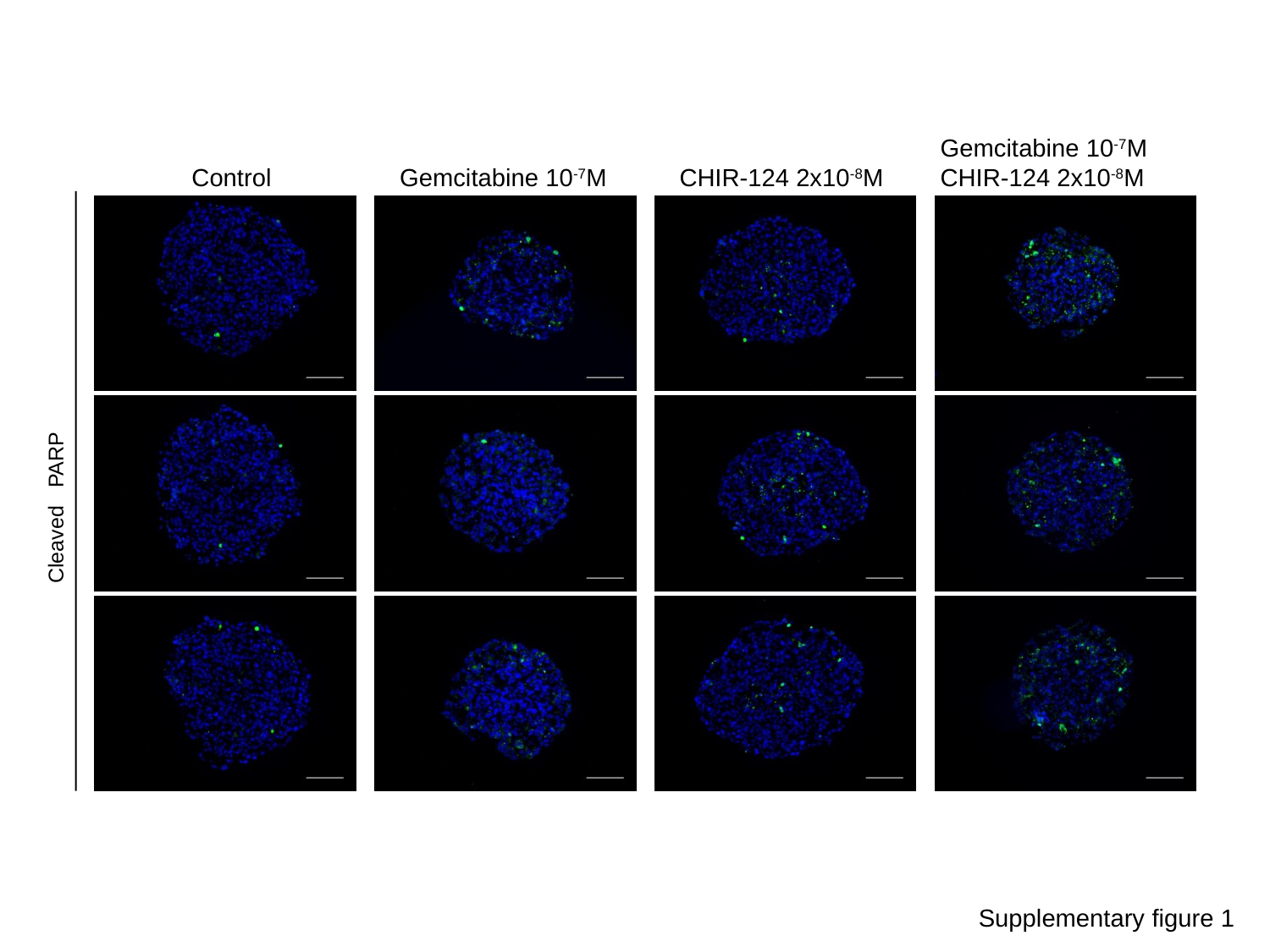

Gemcitabine 10-7M
CHIR-124 2x10-8M
Control
Gemcitabine 10-7M
CHIR-124 2x10-8M
Cleaved PARP
Supplementary figure 1

Supplement: Additional file 1 — Figure S1. Induction of apoptosis upon exposure of Capan-2 spheroid to Gemcitabine and CHIR-124. Analysis was performed as described in Figure 6. Apoptosis was revealed by immunodetection of cleaved form of PARP. Three sections from different spheroids are shown here to illustrate the reproducibility of the observation. The scale bar corresponds to 100 μm. [file 1471-2407-12-15-S1.PPT]
