# Supplementary material for: Multicellular tumor spheroid model to evaluate spatio-temporal dynamics effect of chemotherapeutics: application to the gemcitabine/CHK1 inhibitor combination in pancreatic cancer
Source: BMC Cancer. 2012 Jan 13;12:15. doi: 10.1186/1471-2407-12-15 (PMC3280152; doi:10.1186/1471-2407-12-15)
Supplement: Additional file 2 — Figure S2. Quantification of the induction of apoptosis upon exposure of Capan-2 spheroid to Gemcitabine and CHIR-124. Analysis was performed as described in Figure 6. Apoptosis was revealed by immunodetection of cleaved form of PARP. Spheroid sections were analyzed to quantify the number of cells with cleaved PARP using the FIJI software (measure plugin). The data are expressed as the percentage of spheroid sections displaying 0 to 10, 10 to 20, or more than 20 PARP-C positive cells. [file 1471-2407-12-15-S2.PPT]

## Slide 1
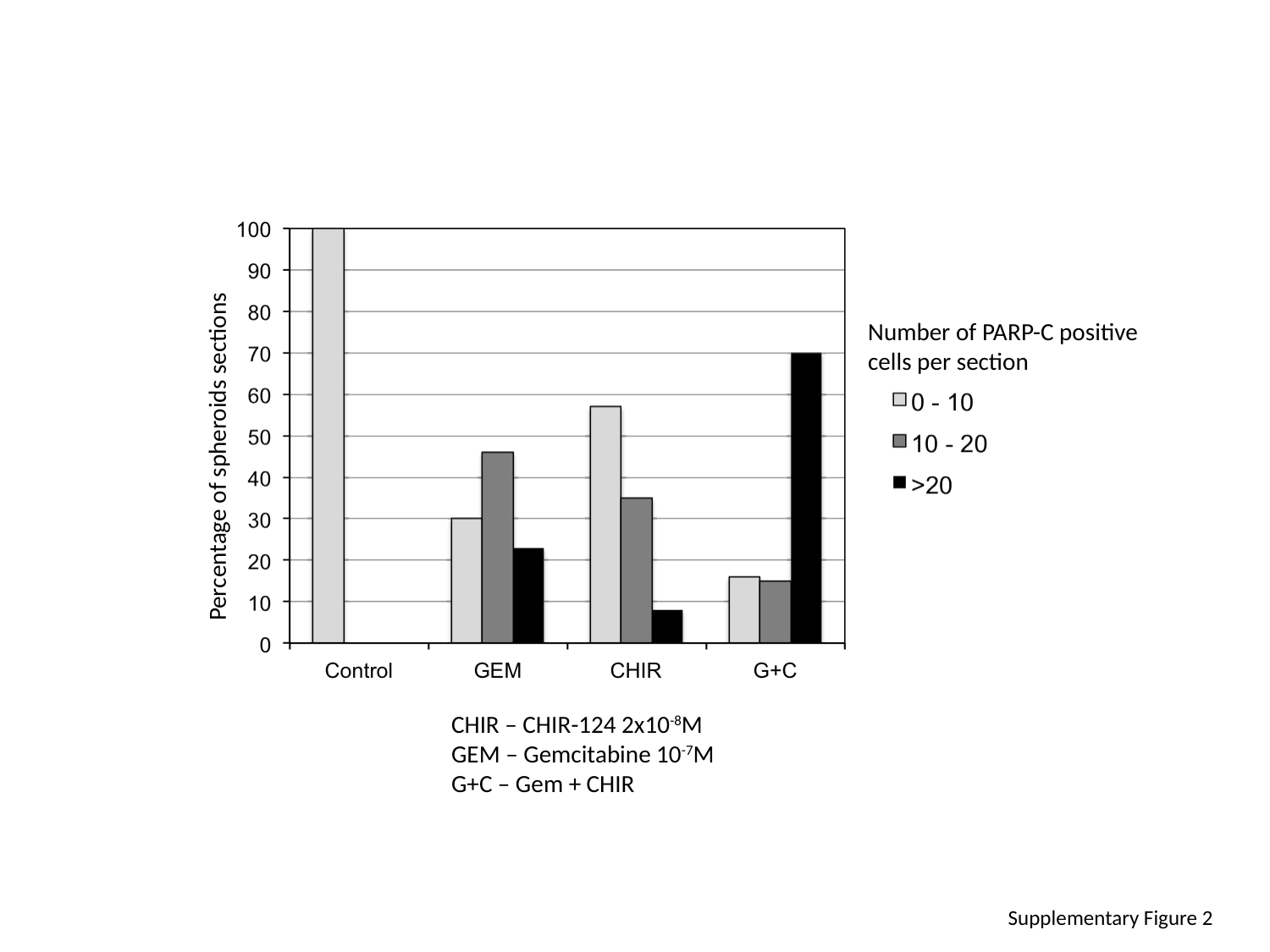

Number of PARP-C positive
cells per section
Percentage of spheroids sections
CHIR – CHIR-124 2x10^-8^M
GEM – Gemcitabine 10-7M
G+C – Gem + CHIR
Supplementary Figure 2
